# Past, present, and future use of phosphorus in Chinese agriculture and its influence on phosphorus losses

**DOI:** 10.1007/s13280-015-0633-0

**Published:** 2015-02-15

**Authors:** Haigang Li, Jian Liu, Guohua Li, Jianbo Shen, Lars Bergström, Fusuo Zhang

**Affiliations:** 1 Center for Resources, Environment and Food Security (CREFS), China Agricultural University, 2 Yuanmingyuan West Road, Haidian District, Beijing, 100193 China; 2 Key Laboratory of Nonpoint Source Pollution Control, Ministry of Agriculture, Institute of Agricultural Resources and Regional Planning, Chinese Academy of Agricultural Sciences, Beijing, 100081 China; 3 Pasture Systems and Watershed Management Research Unit, USDA-Agricultural Research Service, University Park, PA 16802 USA; 4 Department of Soil and Environment, Swedish University of Agricultural Sciences (SLU), P.O. Box 7014, 75007 Uppsala, Sweden

**Keywords:** Phosphorus management zone, Olsen P, Environment, Crop yield, Phosphorus loss

## Abstract

Large inputs of phosphorus (P) in chemical fertilizers and feed supplements since 1978 have improved soil P status in arable land in China, but have also created challenges by increasing P concentrations in manure and exacerbating water quality degradation. Arable land in China can be divided into five management zones based on soil P chemistry, with 15–92 % of arable land having lower P status than the agronomic optimum and 0.3–7.2 % having severe risks of P leaching losses. A scenario analysis of soil P budget and agronomic P demand during 2011–2030 highlighted the great pressure China faces in sustainable P management and the need for drastic changes in current practices. This includes new policies to reduce P supplementation of feed and improved P use efficiency by livestock and programs to expand the adoption of appropriate fertilization, soil conservation, and drainage management practices to minimize P losses.

## Introduction

**Fig. 1:**
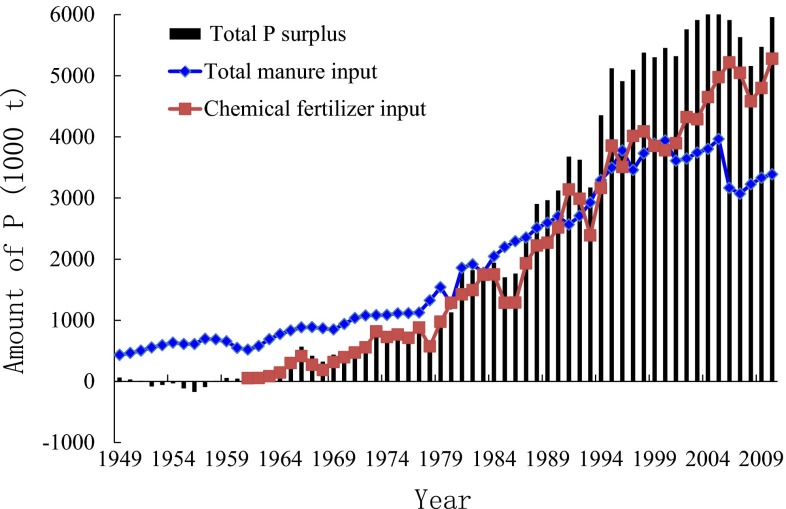
Total P surplus and inputs of P with manure and chemical fertilizers to arable land in China, 1949–2010

During the period 1978–2009, Chinese farmers managed to increase their cereal production from 305 to 531 million tonnes (Mt) and successfully fed the nation’s rapidly growing population, which increased from 960 millions in 1978–1330 millions in 2009 (China Statistics Press 2010a, b). Key agronomic technologies, such as new crop varieties, fertilizers, and pesticides, contributed to this success in agricultural production. Increasing the rate of manure and fertilizer application to land has been critical in achieving the high crop yields, especially since 1978, when the Chinese Reform and Open Policy period began. Consumption of chemical fertilizer phosphorus (P) has increased approximately 100-fold, from 0.05 Mt in 1960, when it was first used, to 5.3 Mt in 2010, with an abrupt increase since 1978 (Fig. 1). Manure P input to Chinese arable land has also increased steadily since 1949, to reach 3.4 Mt in 2010.

It is estimated that less than 20 % of fertilizer P can be used by crops during the growing season in China (Zhang et al. 2008), resulting in a large P surplus in the soil over the long term. In 2010, Chinese arable land had an estimated soil P surplus of 5.9 Mt (Fig. 1). Fortunately, the phosphate reserves in China amount to 3700 million tonnes, a level only exceeded in Morocco (David 2009; Gilbert 2009; China Statistics Press 2010a, b). However, the high consumption of phosphate rock in both fertilizer production (8.8 Mt year^−1^) and animal feed production (383 000 tonnes year^−1^) has been rapidly depleting the high-quality domestic reserves (China Statistics Press 2010a, b; Wang et al. 2011). These reserves will be used up in the coming two decades unless the current extraction rate is modified. Chinese agriculture will then have to face the challenge of high-cost P fertilizer.

Today, there are still large areas of Chinese arable land with Olsen P values lower than 20 mg kg^−1^, the critical fertility level for most field crops (Li et al. 2011). These low-P fields are expected to require large amounts of P fertilizer in the future in order to improve soil fertility. At the same time, excessive P inputs and other inappropriate P management practices can be found in both crop and animal production systems in many areas of China, resulting in severe eutrophication of China’s lakes, rivers, and estuaries (Zhang et al. 2004). Agricultural nonpoint P sources are becoming the major source of P in water (Mao et al. 2005; Cheng et al. 2008; Qian and He 2009). Therefore, the challenge for P management in China is to improve the fertility of low-P soils, increase P use efficiency in animal production systems, and minimize the risk of environmental P losses, especially from soils (even low-P soil) and animal production sectors based on unsustainable P management practices.

In the ‘soil–animal–water’ system, soil P management is of fundamental importance because the soil serves as a sink for manure loads from animal production and is the major source of P losses to water bodies in China. Over the past few decades, several soil P management strategies, including soil-based and plant/rhizosphere-based P management strategies, have been developed by Chinese farmers and agricultural scientists (Li et al. 2011). This paper briefly reviews the status of P in Chinese soils caused by P inputs over the past three decades and describes the basis for a sustainable soil P management strategy for improving soil fertility while reducing environmental P pollution.

## Phosphorus management zones in china

Soil P availability depends on soil properties, the most fundamental of which is pH (Lindsay 1979; Hinsinger 2001). The main phosphate minerals, variscite (AlPO_4_) and strengite (FePO_4_), are known to be stable in highly acidic soil, and they become more soluble as soil pH increases (Lindsay 1979), in contrast to calcium phosphate (Ca_3_(PO_4_)_2_). In the soil pH range 6–6.5, variscite, strengite, and calcium phosphate can coexist. When soil pH is over about 7.9, P solubility increases again, depressing calcium cation formation. Based on soil P chemistry, soils can be classified into three categories: acidic (pH < 6.0), neutral (6.0 ≤ pH < 7.5), and calcareous (pH ≥ 7.5). These categories have been shown to govern inorganic soil P dynamics in 18 long-term fertility experiments in China (Li unpublished data). The major inorganic P fractions are Fe–P and occluded P in acidic soil, Ca_10_–P (hydroxyapatite) in calcareous soil, and a mixture in neutral soil. There are 41 pedogenic categories in China according to the Chinese soil classification system, covering all pH ranges (Zheng et al. 1994).

Because of regional climate variations, a major factor in soil formation (White 2006), soil pH displays roughly a zonal distribution (Zheng et al. 1994). Based on soil pH and climate, five soil P management zones can be distinguished in China, namely Northeast, North, Northwest, Yangtze Plain, and South China. Soils tend to be acidic in South China, calcareous in North and Northwest China, and neutral in Northeast China and Yangtze Plain.

## Critical phosphorus levels for crop production, fertility, and environmental risk in different phosphorus management zones

In China, soil Olsen P value is used to estimate soil P availability to crops. This method employs 0.5 mol L^−1^ NaHCO_3_ to extract labile soil P, and this is considered relatively weak compared with other agronomic extractants (Olsen et al. 1954). Given the uncertainty in crop response and long-term processes that tend to decrease P availability to crops, the agronomic threshold for Olsen P is set slightly higher than the precise critical level identified in fertilizer response curves. The critical value of soil Olsen P for crop yield is defined as that below which crop yield displays a great response to P fertilizer input and above which the response is negligible (Mallarino and Blackmer 1992).

The major crops grown in Northwest, Northeast, and North China are wheat and maize, in Yangtze Plain wheat and rice, and in South China rice (two crops per year). A few studies have been conducted to determine the critical level of soil Olsen P for crop production and P leaching in these zones (Table 1). The optimal range of soil Olsen P has been shown to vary between zones. The lowest recommended level of soil Olsen P for crop production is only 9.0 mg kg^−1^ in Northeast China, and the highest level is 39.0 mg kg^−1^ in South China, where the highest critical level for leaching has also been observed. These variations are due to the chemical properties of the different soils (Shober and Sims 2007).

**Table 1:** Critical level (mg kg^−1^) of soil Olsen P for production of different crops and for P leaching potential in the five P management zones in China

	Northwest	Northeast	North	Yangtze Plain	South
Wheat	16.1	6.6	13.3	13.0	12.7
Maize	14.6	6.6	11.5	–	28.2
Rice	–	–	–	8.7	29.6
Recommended level	21.0	9.0	18.0	17.0	39.0
Leaching level	36.9	51.6	51.0	76.1	78.2

Re-calculated data from Bai et al. (2013), Tang et al. (2009) and Zhong et al. (2004)

Phosphorus in the soil can be lost to water through two transport pathways, namely surface runoff and erosion by overland flow and vertical leaching to drainage depth. Overland P losses are a major concern in the large areas of China vulnerable to soil erosion, such as the mountains and uplands of West China and the Loess Plateau in North China, as well as the Three Gorges region crossing about 20 counties (Chen et al. 2002; Zhang et al. 2011a; Wu et al. 2012). Overland P losses are also a concern in regions with a flat landscape but extensive water networks, e.g., the Lake Taihu region (Guo et al. 2004; Peng et al. 2011). Phosphorus leaching losses are a major concern in areas with intensive arable soils receiving high inputs of mineral P fertilizer and/or animal manure, which can be found in all five of China’s P management zones. Hesketh and Brookes (2000) identified a change point between soil Olsen P and soil CaCl_2_–extractable P that was consistent with the Olsen P threshold identified for P losses in subsurface drains. Below the change point soil CaCl_2_–P was negligible, but above the change point, it increased abruptly. Many studies have since reported change points between various soil P extracts, as well as in the relationship of soil P with P concentrations in surface runoff or leachate (McDowell et al. 2001; Kleinman et al. 2007). Studies in China have confirmed that soil CaCl_2_–extractable P is closely related to P concentrations in leachate (Zhang et al. 2005), and the relationship between soil Olsen P and CaCl_2_–extractable P as identified by Hesketh and Brookes (2000) has been relatively well validated (Qin et al. 2010; Bai et al. 2013). Thus, the Olsen P method is used by many Chinese researchers as an indicator of off-site P pollution potential. Given the fact that China has a large arable land area covering a great diversity of topography, which can strongly determine the magnitude of P losses by overland flow (Sharpley et al. 2003), the potential risk of P losses based on soil P status and budget estimated in the present study is mostly relevant for P leaching losses.

## Phosphorus status and budget in arable land

**Fig. 2:**
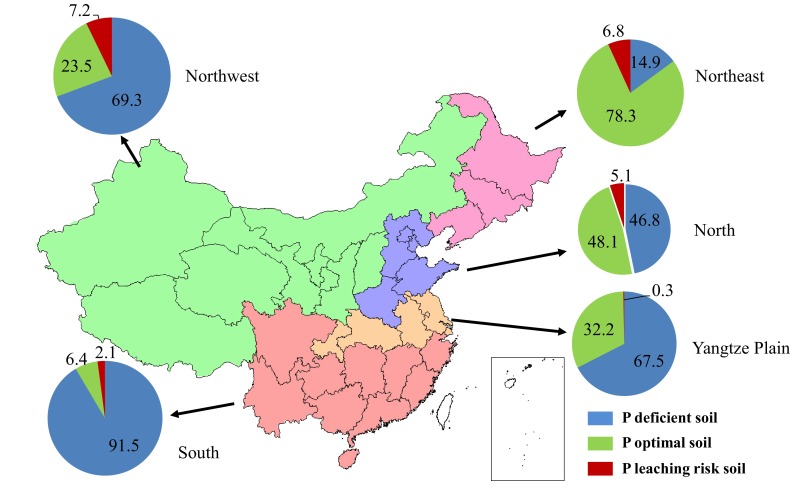
Proportions (%) of arable land with different soil Olsen P status in the Northeast, Northwest, North, Yangtze Plain, and South P management regions of China

Soil Olsen P status in different P management zones of China in 2009 is shown in Fig. 2. The relative proportions of P deficient (Olsen P < recommended level), P-optimal (recommended level ≤ Olsen P < leaching level), and P leaching risk (Olsen P ≥ leaching level) arable land vary widely in the different P management zones. In Northwest China, Yangtze Plain and South China, P-deficient land still accounts for more than 50 % of total arable area. In South China in particular, the Olsen P value in more than 90 % of arable soils is lower than that recommended for crop production (39 mg kg^−1^). Arable land with optimal soil Olsen P comprises 48 % of the total arable area in North China and 78 % in Northeast China, which together contribute 40 % of total crop production in China (China Statistics Press 2010a, b). All five P management zones have some soils with a P leaching risk, with the proportion varying from 0.3 to 7.2 % of the total arable area.

**Fig. 3:**
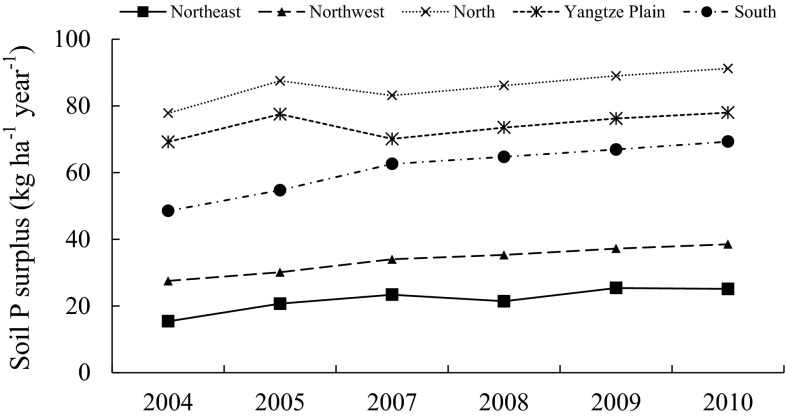
Soil P surplus (inputs minus removal with crop) in the five P management zones in China, 2004–2010

Inputs of large amounts of P fertilizer to Chinese soils from 1980 to 2007 resulted in an average surplus of more than 240 kg P ha^−1^ (Li et al. 2011). The soil P surplus increased over time and contributed to the rapid improvement in soil fertility from 1980 onwards. During 2004–2010, mean soil P surplus reached 86 kg ha^−1^ year^−1^ in North China (Fig. 3). This is slightly higher than the value previously reported by Vitousek et al. (2009) for the same region. Northeast China had the lowest mean soil P surplus during this period (22 kg ha^−1^ year^−1^) (Fig. 3), but the surplus was still much higher than in e.g., Western Kenya (1 kg ha^−1^ year^−1^), Midwest USA (9 kg ha^−1^ year^−1^), and Denmark (11 kg ha^−1^ year^−1^) (Maguire et al. 2009; Vitousek et al. 2009). Manure P input has been playing an ever-increasing role in the soil P surplus in Chinese soils over recent decades (Table 2). From 1986 to 2009, animal production increased almost sixfold in China (China Statistics Press 2000). Manure P applications amounted to 3.8 Mt in 2003 (Wang et al. 2006), but had increased to 8.4 Mt by 2010. The average manure P load to arable land was 70 kg ha^−1^ year^−1^ in the whole country. This is much higher than the rate permitted in, for example, the Netherlands (35 kg ha^−1^ year^−1^) (Oenema et al. 2004) or Sweden (22 kg ha^−1^ year^−1^) (SBA 2010). Even in Northwest China, the zone with the lowest manure P loads, the annual P load has already reached 36 kg ha^−1^, while it is more than 80 kg ha^−1^ in both North China and South China (Table 2).

**Table 2:** Area of arable land, manure production, and manure P loading to arable land in the five soil P management zones in China, 2010

P management zone	Arable land (×10^6^ ha)	Manure production (×10^6^ kg)	Manure P load (kg ha^−1^)
Northeast	21.5	862.0	40.2
Northwest	26.0	926.3	35.6
North	22.4	1935.0	86.3
Yangtze Plain	17.6	1316.3	74.6
South	34.1	3410.0	99.9

In order to meet the increasing demand for food from the rapidly growing population and the dietary shift from vegetable to animal products in China, crop production has to reach 580 Mt by 2030, with a 2 % increase annually from now on (Wang et al. 2011; Zhang et al. 2011b). This poses an even greater challenge for rational management of soil P in the future. In the P-deficient arable soils, build-up of the soil P pool is necessary to improve soil fertility and crop yields. However, for the soils with a high risk potential of P losses (approximately 4 % of total arable land), soil P must be appropriately managed to minimize losses and the associated impact on water quality.

## Phosphorus losses from different phosphorus management zones

At present, agriculture is the largest source of P loads to many waters in China, as technologies and experience of measures for combating other point sources of P have improved greatly in recent decades. For example, agriculture is estimated to contribute 38–90 % of the gross P load to Lake Taihu, 40–52 % to Lake Chaohu, and 30–60 % to Lake Dianchi (Chen et al. 2006). These ‘Three Lakes’ are among the most severely eutrophied water bodies in China and have received great attention in research and pollution control work (Qu and Fan 2010). According to estimates by China’s First National General Census of Pollution Sources, the whole country emitted a total of 4.23 × 10^5^ tonnes P to the environment (mainly all kinds of waters) in 2007 (CPSC 2011). Of this, 67 % came from the agriculture sector, including 26 % from crop production, 38 % from animal production, and 3 % from aquaculture directly in waters. On average, for the total arable area in China, an estimated total amount of 2.2 kg P ha^−1^ was lost from crop and animal production systems. This rate of P loss is quite high compared with that estimated for many developed Western countries, for example, 0.4 kg ha^−1^ year^−1^ in Sweden and 0.5 kg ha^−1^ year^−1^ in the United Kingdom and the Republic of Ireland (Ulén et al. 2007). Considering the fact that eutrophication is still occurring in many waters in these Western countries despite the currently low agricultural P losses, the way ahead for China in reducing agricultural sources of P losses and combating eutrophication could be long and difficult.

**Fig. 4:**
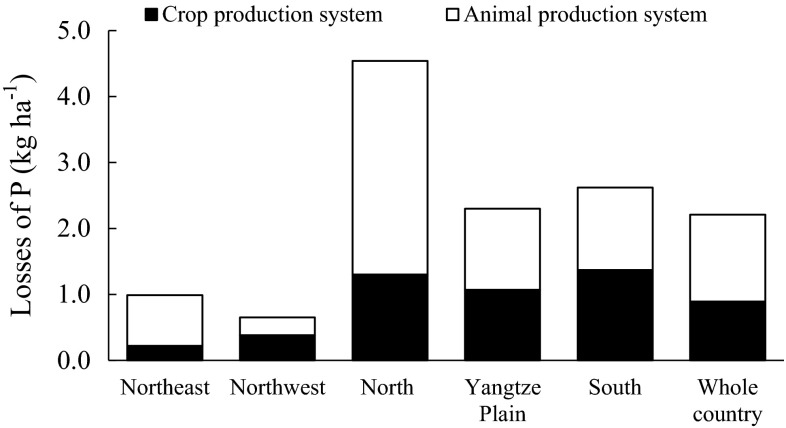
Losses of P from crop and animal production systems in the five P management zones in China and in the whole country in 2007 (based on estimates by CPSC 2011)

The estimated P losses from arable land in China vary greatly with P management zone (Fig. 4). Overall, North China, Yangtze Plain, and South China have greater P losses than the Northeast and Northwest zones. This reflects the fact that the former three zones have a much larger soil P surplus than the latter two (Fig. 3). The large soil P surplus in North China, Yangtze Plain, and South China is the result of dense population and intensive crop and animal production in these zones. It should be noted that P losses from animal production systems are often larger than those from crop production systems. In particular, animal production systems contribute as much as 70 % of total P losses in the North China zone and 80 % of those in Northeast China. A number of studies across the world have demonstrated that application of manure can cause similar or even higher risks of P losses from soils than use of mineral P fertilizers (Shepherd and Withers 1999; Liu et al. 2012a). In China, animal manure is commonly applied in vegetable production, but farmers pay little attention to the potential risk of P losses caused by manure. As a result, the rate of P application is always extremely high when manure is applied, particularly when it is used in addition to mineral fertilizers. Xue et al. (2013) demonstrated that manure application during 8–15 years significantly increased the risk of P losses from Chinese soils. Due to long-term P application, the soil Olsen P in vegetable fields is typically twofold to tenfold higher than that in nearby crop fields in Beijing (Su et al. 2002).

## Management strategies for soil phosphorus and phosphorus losses

### Build-up and maintenance approach to soil P management

The problems in P use, including the common over-application of fertilizer and manure P on arable land, need to be dealt with using an integrated management strategy. Regarding soil P, both soil P availability and P inputs from fertilizers and manure should be precisely determined in order to improve P fertility in low-P soils, maintain P level in soils with an optimal P content, and reduce the risk of P losses in soils with a high P content. In the animal production sector, strict standards on P feeding similar to those enforced in the developed countries and other technologies to improve P use efficiency by animals should be adopted, in order to reduce the P content in manure and alleviate manure P load stress in arable land. At the same time, sources of P losses to water from soil erosion, runoff from high P soils, and manure in animal production should be minimized, e.g., by reducing soil P concentrations to below the environmental risk level and recycling manure to arable land.

**Fig. 5:**
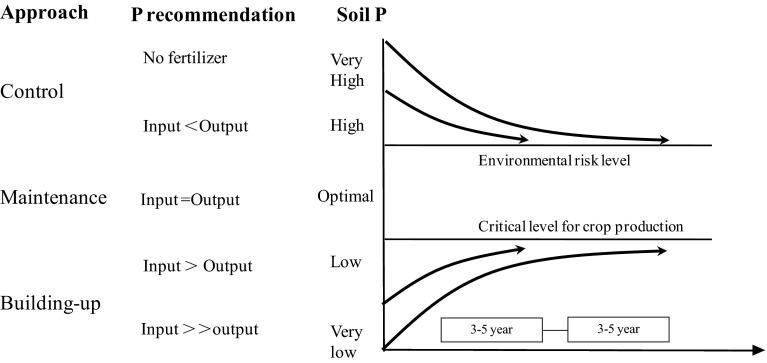
Theoretical model of the Build-up and Maintenance approach used in soil P management in China (adapted from Li et al. 2011)

A Build-up and Maintenance approach based on a soil P budget is one of the soil-based P management strategies adopted by the National Soil Testing and Fertilizer Recommendation Program in China since 2005 (Li et al. 2011). The principles of this approach are illustrated in Fig. 5. Depending on whether soil Olsen P level is high, low, or optimal, P fertilizer inputs can be less than, more than, or equal to crop P removal, respectively. This approach can also be used to predict the need for P in order to improve soil Olsen P to the optimal level in P-deficient soil and to predict the time required to reduce high P to the optimal level. Long-term fertility experiments have supplied the necessary data to build the relationship between change in soil Olsen P and soil P budget in different P management zones of China. The increase in soil Olsen P caused by a P surplus of 100 kg ha^−1^ year^−1^ in the form of only chemical P fertilizer inputs is 6 mg kg^−1^ in Northeast China, 2 mg kg^−1^ in Northwest China, 3 mg kg^−1^ in North China, 4 mg kg^−1^ Yangtze Plain, and 3 mg kg^−1^ in South China (Cao et al. 2012). These variations in the rate of change in soil Olsen P in different P management zones are primarily due to differences in soil physico-chemical properties, especially soil pH (Lindsay 1979; Blake et al. 2000). When P fertilizer and manure are added to soil together, it has been shown that the increase in soil bioavailable P is more than the sum of the increase from either input singly (Toor and Bahl 1997; Reddy et al. 1999). Studies in China show that the ratio of increase in soil Olsen P between chemical P fertilizer plus manure together and chemical P fertilizer inputs alone is 1.88, 1.03, and 1.31 in calcareous, neutral and acidic soils, respectively (Li, unpublished data). This can be explained by desorption of P anions from soil minerals by organic compounds in manure (Shen et al. 2011).

### Strategies to mitigate P losses from agricultural production

Appropriate management of P sources is essential to reduce P losses from agricultural soils (Table 3). Reduced fertilizer or manure P application has been demonstrated to efficiently reduce P losses from agricultural soils in many studies worldwide (see e.g., review by Kleinman et al. 2011). In China, reducing manure P application by 50 % has been reported to reduce soluble P in surface runoff from cabbage fields by 33 % (Liu et al. 2007) and to reduce lateral P seepage losses from organic rice production systems by 53 %, without affecting crop yield (Liang et al. 2013). When fertilizer or manure P is applied to a deep soil layer or incorporated into the soil, the concentrations of both dissolved and particulate P in surface runoff can be substantially reduced compared with surface application (Wang et al. 2013). Elsewhere, Uusi-Kämppä and Heinonen-Tanski (2008), and Kleinman et al. (2002) have found that injection of manure into the soil can reduce P runoff losses by up to 80 % compared with surface spreading. Similarly, the risk of P leaching can be reduced by half when manure is mixed into the soil (Liu et al. 2012b). In rice production, when heavy rainfall and associated surface runoff occur a few days after P application, losses of P can be very high (Zhang et al. 2002). Therefore, management strategies should cover the period between P application and flooding events, for example, by applying fertilizer and manure outside the main rainy season, in order to reduce P losses (Wang et al. 2001; Zhang et al. 2002). In particular, application of manure under wet conditions should be avoided, since it causes an even higher risk of P losses from rice fields than mineral P application (Wang et al. 2001; Zhang et al. 2002).

**Table 3:** Strategies to mitigate P losses from agricultural production in China

Strategy	Aim	Measure	Scale	References
Management of P sources	To avoid accumulation and saturation of P in soils	Reduced rate of fertilizer and manure P applications	Field	Liu et al. (2007) and Liang et al. (2013)
	To reduce availability of P sources	Deep application or incorporation of P into the soil	Field	Zeng et al. (2008) and Wang et al. (2013)
	To apply P at the right time	Apply P, in particular manure P, out of rainy season in rice production	Field	Wang et al. (2001) and Zhang et al. (2002)
Use of industrial (by-)products	To treat wastewater from farms with intensive animal production	Treat wastewater with aged refuse	Farm	Zhao and Shao (2005)
	To retain P in soil	Apply polyacrylamide, lime and gypsum	Field	Jiang et al. (2010)

In China, there is growing interest in using industrial products or by-products to reduce P losses from agricultural systems. For instance, P in wastewater produced on intensive animal farms can be efficiently absorbed by aged material excavated from municipal solid waste landfills (Zhao and Shao 2005). Use of one tonne of such material daily can treat 300 L livestock wastewater containing 24–100 mg P L^−1^ (Zhao and Shao 2005). Incorporation of polyacrylamide into the soil can significantly reduce P losses via surface runoff and leaching compared with an unamended control (Jiang et al. 2010). For runoff P losses, application of lime (CaCO_3_) or gypsum (CaSO_4_·2H_2_O) can also help decrease total P losses (Jiang et al. 2010).

## Scenario analysis to 2030

### Soil P budget 2011–2030

Based on the current P budget (C) or the Build-up and Maintenance approach (B), in combination with or without manure recycling (M), four scenarios (C, CM, B, and BM) were developed in this study to explore soil P status and potential demand for P fertilizer in 2030. Scenarios C and CM assumed that the soil P surplus present in 2010 persisted during the period 2011–2030, while scenarios B and BM assumed that the Build-up and Maintenance approach was adopted during 2011–2030. As regards manure recycling, scenarios C and B assumed that the current rate of manure recycling (43.5 %; Bao et al. 2003) was maintained. When manure P production was larger than the P demand, maximum manure P recycling was assumed to be equal to the P demand. Scenarios CM and BM instead assumed preferential manure recycling (max. rate 100 %) to arable land instead of chemical P fertilizer application. Manure P production was assumed to maintain the level reported for 2010.

### Demand and input of P in scenarios

According to the scenarios, the accumulated amount of manure P in China in the period 2011–2030 was 175.9 Mt (Table 4). South China, which accounted for 40.4 % of the total manure P in China, had the largest accumulated amount of the five P management zones. This can be explained by the large number of intensive livestock farms in South China (China Statistics Press 2010a, b). During the 20-year study period, the total demand for P in Chinese arable land was 203 Mt in scenarios C and CM. The ratio of chemical P input to manure P input in scenario C was 1.5 in Northeast China, 2.0 in Northwest China, 1.9 in North China, 2.0 in Yangtze Plain, and 1.0 in South China. The non-recycled 95.4 Mt manure P will most likely enter the environment. When manure P was the preferential input to arable land (scenario CM), the total demand for chemical P decreased to 33.4 Mt, which was only 27.2 % of the chemical P input needed in scenario C. The total manure P left was 6.3 Mt in scenario CM, which is much lower than the excess (95.4 Mt) in scenario C. Manure P could be 100 % recycled in Northeast, Northwest China, North China, and Yangtze Plain. When the Build-up and Maintenance approach was assumed to be adopted in soil P management (scenarios B and BM), the P demand was only 65.7 Mt, which is 32.4 % of that in scenarios C and CM. The consumption of chemical P fertilizer was 0.2 Mt and of manure P 65.5 Mt in scenario B. In scenario BM, no chemical P was needed for improvement of soil fertility, as manure P was sufficient to meet the P demand in all P management zones. Because of limited P demand in scenarios B and BM, a large amount of manure P (110.4 Mt in scenario B and 110.2 Mt in scenario BM) was not recycled to arable land. Under these conditions, China would have to face the challenge of environmental pollution caused by excess manure P, unless this P is used in other ways.

**Table 4:** Phosphorus budget (Mt) according to the four different scenarios in the five P management zones in China, 2011–2030

	Northeast	Northwest	North	Yangtze Plain	South	Total
Total manure P	17.7	19.4	40.3	27.5	71.0	175.9
*Scenario C*						
Total P demand	19.6	27.3	53.8	37.6	64.7	203.0
Chemical P input	11.7	18.4	35.4	25.0	32.2	122.7
Manure P input	8.0	9.0	18.4	12.6	32.5	80.5
Manure P non-recycled	9.7	10.4	21.9	14.9	38.5	95.4
*Scenario CM*						
Total P demand	19.6	27.3	53.8	37.6	64.7	203.0
Chemical P input	1.9	7.9	13.5	10.1	0	33.4
Manure P input	17.7	19.4	40.3	27.5	64.7	169.6
Manure P non-recycled	0	0	0	0	6.3	6.3
*Scenario B*						
Total P demand	8.2	8.8	13.7	11.6	23.4	65.7
Chemical P input	0.2	0	0	0	0	0.2
Manure P input	8.0	8.8	13.7	11.6	23.4	65.5
Manure P non-recycled	9.7	10.6	26.6	15.9	47.6	110.4
*Scenario BM*						
Total P demand	8.2	8.8	13.7	11.6	23.4	65.7
Chemical P input	0	0	0	0	0	0
Manure P input	8.2	8.8	13.7	11.6	23.4	65.7
Manure P non-recycled	9.5	10.6	26.6	15.9	47.6	110.2

Scenario C assumed that the soil P surplus present in 2010 persisted during the period 2011–2030, and the current rate of manure recycling was maintained. Scenario CM assumed that the soil P surplus present in 2010 persisted during the period 2011–2030 and preferential manure recycling to arable land instead of chemical P fertilizer application. Scenario B assumed that the Build-up and Maintenance approach was adopted during 2011–2030, and the current rate of manure recycling was maintained. Scenario BM assumed that the Build-up and Maintenance approach was adopted during 2011–2030 and preferential manure recycling to arable land instead of chemical P fertilizer application

### Soil P status in different P management zones of China in 2030

**Fig. 6:**
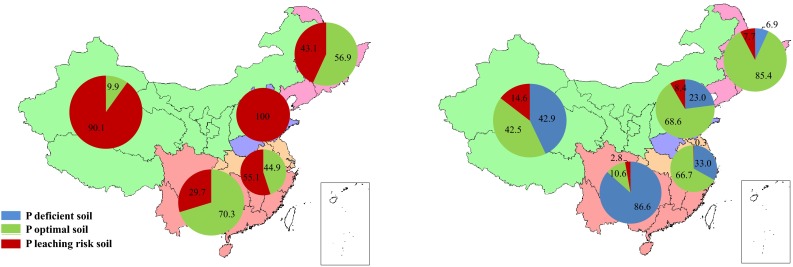
Predicted soil Olsen P status in the five P management regions in China in 2030 according to (*left*) scenarios C and CM; and (*right*) scenarios B and BM (For explanation of scenarios see Table 4)

In scenarios C and CM, only a small area of arable land was P deficient in 2030 and this was only in Northwest China (Fig. 6a). A total of 43.1–100 % of arable land had a potential risk of P leaching in Northeast China, North China, and Yangtze Plain. In South China, soil Olsen P in most arable land was optimal for crop yield, but 29.7 % of arable land had a potential risk of P leaching. Thus, the current P input rate will cause an unimaginable environmental disaster in the future and will definitely not be sustainable for future agriculture in China. Fortunately, the Chinese government and scientists have realized this problem and have developed and implemented a new soil P management strategy—the Build-up and Maintenance approach.

In scenarios B and BM, after adoption of the Build-up and Maintenance approach, the proportion of arable land with a P leaching risk substantially decreased in all five P management zones (Fig. 6b). Soil Olsen P reached an optimal level in 68.6 % of arable soils in North China, 85.4 % in Northeast, and 66.7 % in Yangtze Plain by 2030. In contrast, 86.6 % of the arable land in South China still needed improvement of soil fertility by excess chemical P fertilizer and manure P inputs.

It should be noted that the rate of reduction in P losses brought about by a specific source management practice often depends strongly on the magnitude of soil P inputs, among other factors. The reduction in P losses is not always linearly correlated with the reduction in P inputs due to the complexity of soil physical and chemical properties. At high rates of P inputs, P losses can be reduced relatively easily by rational management of P sources; but when P inputs are small, the reduction in P losses is often marginal and shows great variability. Moreover, when the P applied with fertilizer and manure is bound in the soil, it constitutes a long-term source of P losses. Consequently, it is difficult to predict changes in P losses over the long term.

## Perspectives

The remaining high-grade phosphate reserves (≥30 % P_2_O_5_) in China correspond to 231 Mt P (Yi unpublished data). At the extraction rates reported in 2009 (8.8 Mt; China National Chemical Information Center 2010), these reserves are predicted to last for only 25 years. Therefore, China needs a sustainable P management strategy for the soil–animal–water system, with the focus on soil. This includes management of fertilizer inputs to promote crop yields, manure P recycling, and reducing P losses from soil and manure. The Build-up and Maintenance approach is now being adopted in soil P management in China. However, although it is practical and feasible for general farmers, it is not sufficient to ensure the sustainability of soil P, as the crop/rhizosphere potential and precise manure management are basically ignored.

By exploiting crop/rhizosphere potential to improve P use efficiency, the agronomic optimal soil P level can be lowered, while the pressure on limited P rock reserves and the environment can also be alleviated. It is very important to achieve high crop yields in low-P soils, but it takes a long time to improve soil fertility by P inputs. Liao et al. (2001, 2004) successfully selected a common bean cultivar with shallow roots that matched the P distribution in soil horizons, and use of this type of bean has been extended in South China. Chinese farmers have been using intercropping to improve crop yield in low-nutrient soils for two thousand years (Li et al. 2001). The yield increment by intercropping is usually attributed to rhizosphere interactions between different plant species (Li et al. 2007, 2013). In intensive farming, nutrient uptake and growth of maize can be improved by rhizosphere process manipulation (Jing et al. 2010). Therefore, Chinese scientists should focus on improving or developing techniques for manipulation of rhizosphere processes for sustainable soil P management.

Besides further exploration and more intensive exploitation of phosphate rock resources, maximizing recovery of the P flows in the loop from the fertilizer applied to arable land to the food on the table will increase the life expectancy of phosphate rock reserves (Cordell et al. 2009). It is estimated that more than 40 % of the P mined globally can be recovered with complete reuse of animal wastes (Rittmann et al. 2011). Phosphorus recovery measures include returning crop straw to the field and composting food waste (Cordell et al. 2009). Many provinces in China started to return straw to the field several years ago, especially Hebei province, where the rate of straw return has been nearly 100 % in recent years because of government support. However, straw return is still facing several problems in China, such as high labor costs, difficulty in disease control, and shortage of machinery. In order to extend straw return to the whole of China, the Chinese government must provide policy and financial support to farmers.

In a study in Brazil, Lopes (1998) analyzed several strategies differing in the time required to build up P in the soil and showed that farmers were willing to adopt strategies which took four to 6 years to build up soil P without too much investment cost and crop yield losses. In China, soil P in a large proportion of arable land will still be below the optimal level in 21 years according to scenarios B and BM. Under the current land policy in China, farmers have the right to manage their arable land for 30 years. After that, it is possible for arable land to be reallocated. It appears reasonable that Chinese farmers do not want to invest too much in their temporary land. If the government develops a policy to encourage farmers to improve the fertility of their land, the build-up rate of soil P in the Build-up and Maintenance approach can be increased.

Many mitigation strategies tested in China have been designed not only for P, but also for N and other pollutants. In practice, this makes it difficult to decide whether to use a strategy based mainly on its efficiency in reducing P losses. Some of the current practices widely used to mitigate diffuse pollution from agriculture may not be the best practice for P but work for other pollutants, and vice versa. Such practices need to be refined for areas, where P is a primary concern. Moreover, current tests on mitigation strategies cover only a small range of practices, and testing definitely needs to be expanded to a wider range of practices and to be more thorough. Use of simulation models to test mitigation options has generated wide interest among Chinese researchers, but these models are primarily developed for Europe and North America and they have not been appropriately calibrated and validated for Chinese conditions (Ongley et al. 2010). Therefore, the applicability of these models in China is currently limited and more work is needed.

After analyzing the P footprint of China’s food chain in this study, we concluded that the current P utilization pattern is not sustainable for either food security or environmental safety in China. This is because of considerable amounts of P losses from soil erosion, runoff and leaching, and insufficient recycling of animal and human excreta (Wang et al. 2011). At the current rate of manure P excretion by animals, arable land was unable to accommodate all of the manure P in any of the four scenarios tested. The concentration of P in manure ranges from 7.5 to 2.4 g kg^−1^ in China (Li et al. 2014), which is comparable to that in the USA (Dou et al. 2003; Turner and Leytem 2004). In contrast, P use efficiency in animal production systems is only 6 % in China (Li unpublished data). Thus, there is great potential to reduce total manure P excretion by improving P use efficiency in animal production. In order to alleviate the pressure of food security and reduce P losses to the environment, China needs to (1) modify the Build-up and Maintenance approach to also exploit the crop/rhizosphere potential; and (2) adopt appropriate management practices to minimize P losses via soil erosion, runoff, and leaching and to optimize P inputs in animal production and maximize manure recycling during crop production.
